# Δ^9^-Tetrahydrocannabinol exposure shifts eosinophil and macrophage transcriptional programs toward an anti-inflammatory phenotype in helminth infection

**DOI:** 10.1093/jleuko/qiag131

**Published:** 2026-09-24

**Authors:** Jennell Jennett, Martin Olmos, Ka Man Lam, Sarah Midou, Nicholas V DiPatrizio, Meera G Nair

**Affiliations:** Division of Biomedical Sciences, School of Medicine, University of California Riverside, 900 University Avenue, Riverside, CA 92521, United States; Division of Biomedical Sciences, School of Medicine, University of California Riverside, 900 University Avenue, Riverside, CA 92521, United States; Division of Biomedical Sciences, School of Medicine, University of California Riverside, 900 University Avenue, Riverside, CA 92521, United States; Division of Biomedical Sciences, School of Medicine, University of California Riverside, 900 University Avenue, Riverside, CA 92521, United States; Division of Biomedical Sciences, School of Medicine, University of California Riverside, 900 University Avenue, Riverside, CA 92521, United States; Division of Biomedical Sciences, School of Medicine, University of California Riverside, 900 University Avenue, Riverside, CA 92521, United States

**Keywords:** eosinophils, helminth infection, macrophages, THC, type 2 inflammation

## Abstract

Cannabis use is increasing globally, yet the immunological effects of Δ^9^-tetrahydrocannabinol, the main intoxicating component of cannabis, remain incompletely understood. Given prior evidence that endocannabinoid signaling influences helminth immunity and type 2 inflammation, we investigated how sustained Δ^9^-tetrahydrocannabinol exposure alters immune responses to the helminth *Nippostrongylus brasiliensis*, which infects the lung and small intestine of mice. C57BL/6J mice were treated with Δ^9^-tetrahydrocannabinol (5 mg/kg/d) or vehicle for 14 d prior to helminth infection and assessed for parasite burden, innate immune cell and T cell responses, and transcriptional changes in lung eosinophils and macrophages. Δ^9^-Tetrahydrocannabinol exposure did not significantly alter infection-associated weight loss or helminth burden; however, Δ^9^-tetrahydrocannabinol selectively restrained infection-induced circulating eosinophils and monocytes while increasing regulatory T cells. T cell activation assays showed reduced TNFα and IFNγ secretion in splenocytes from Δ^9^-tetrahydrocannabinol-treated infected mice. Bulk RNA sequencing showed that Δ^9^-tetrahydrocannabinol shifted lung eosinophils and CD11c^+^ lung macrophage-enriched cells from inflammatory, fibrotic, and costimulatory pathways toward stress and metabolic-adaptive transcriptional programs. Within the infected macrophage-enriched population, Δ^9^-tetrahydrocannabinol reduced CD80 expression while increasing MHC class II and antigen presentation-associated genes, suggesting a potential shift in macrophage-mediated T cell activation. Consistent with altered inflammatory and tissue remodeling-associated programs, immunofluorescent staining showed that Δ^9^-tetrahydrocannabinol mitigated infection-associated loss of lung collagen. Collectively, these findings indicate that Δ^9^-tetrahydrocannabinol reshapes the immune response to helminth infection by restraining innate and T cell effector responses while altering lung eosinophil and macrophage activation programs.

## Teaser

THC reshapes helminth-induced type 2 inflammation by restraining inflammatory leukocyte responses and reprogramming lung eosinophils and macrophages.

## 1. Introduction

Cannabis is one of the most used psychoactive substances in the United States. In 2022, an estimated 61.9 million people aged 12 yr or older reported past-year cannabis use, corresponding to 22.0% of the population in this age group. Cannabis use was highest among young adults aged 18 to 25, with 38.2% reporting past-year use.^1^ As cannabis use increases with expanding medical and recreational legalization, there is a growing need to understand how cannabis-derived compounds affect immune function. Δ^9^-Tetrahydrocannabinol (THC) is the primary intoxicating component of cannabis and acts as a partial agonist of cannabinoid receptors.^2^ Although THC is commonly studied for its neurological and behavioral effects, cannabinoid receptors and endocannabinoid pathways are also involved in immune regulation, raising important questions about how THC exposure alters inflammatory responses.^3,4^

The endocannabinoid system has emerged as an important regulator of host immunity during infection and inflammation. In helminth infection, both host- and helminth-derived endocannabinoids are generated and can influence host immune responses and parasite burden.^5^ Human studies have also suggested a relationship between cannabis use and helminth infection. Among Aka foragers of the Congo Basin, cannabis use was highly prevalent, and levels of urinary Δ^9^-tetrahydrocannabinol-11-oic acid (THC-COOH, the primary metabolite of THC) were negatively associated with parasite infection and reinfection.^6^ However, this study evaluated whole-cannabis exposure in a human population and could not determine whether THC directly altered parasite burden, host immunity, or both. Together, these findings suggest that cannabinoid pathways intersect with anti-helminth immunity, but the specific effects of THC on host immune responses remain unclear.

Infection with *Nippostrongylus brasiliensis*, a rodent hookworm, is a well-established murine model of helminth infection and type 2 inflammation, inducing eosinophilia, macrophage activation, Th2 cytokine responses, and lung tissue remodeling.^7–9^ This model provides a controlled system to determine whether THC changes the magnitude or quality of immune responses during type 2 infection and inflammation. Prior work from our group showed that inhibition of peripheral cannabinoid receptor subtype 1 during *N. brasiliensis* infection increased airway eosinophilia and worsened pulmonary pathology, suggesting that cannabinoid signaling can restrain eosinophilic inflammation in this setting.^10^ Considering that THC is a partial cannabinoid receptor agonist, these findings raise the possibility that THC exposure may alter endocannabinoid signaling at cannabinoid receptors and ensuing inflammatory responses during helminth infection-induced type 2 immunity.

THC has also been shown to regulate immune pathways relevant to inflammatory activation. In activated human T cells, THC suppresses proliferation, reduces IFNγ production, and alters Th1/Th2 cytokine balance.^11^ In the myeloid compartment, THC has been reported to inhibit macrophage chemotaxis and alter macrophage differentiation through reactive oxygen species-dependent mechanisms.^12–15^ These findings suggest that THC can regulate both adaptive and innate immune pathways. However, whether THC exposure alters the balance between immune activation, inflammatory restraint, and tissue remodeling during type 2 inflammation remains poorly understood.

Despite these observations, critical gaps remain. First, it is unclear whether THC alone is sufficient to alter parasite burden in a controlled helminth infection model, especially given human studies linking cannabis consumption to reduced helminth burden.^6^ Second, it is unknown whether THC alters type 2 inflammation by reducing immune cell accumulation, changing inflammatory function, or modifying immune cell activation states. In addition, the consequences of THC-associated immune modulation for lung tissue repair following infection-induced injury remain unclear.^9,16^ To address these gaps, we used *N. brasiliensis* infection as a controlled model of helminth infection and type 2 inflammation to evaluate how THC exposure affects parasite burden, innate and T cell immune responses, and overall outcomes in infection-induced lung tissue injury.

## 2. Materials and methods

### 2.1. Mouse studies

All animal procedures were approved by the University of California, Riverside Institutional Animal Care and Use Committee (AUP#48). Six-week-old male C57BL/6J mice were maintained under standard vivarium conditions with ad libitum chow and water. THC was stored, prepared, and administered by authorized personnel in accordance with DEA controlled substance requirements and UCR institutional procedures. THC was dissolved in a vehicle consisting of 7.5% DMSO, 7.5% Tween 80, and 85% sterile saline. Mice were injected intraperitoneally daily with THC (5 mg/kg/d; generously donated by the National Institute on Drug Abuse Drug Supply Program) or vehicle for 14 d prior to infection. The 5 mg/kg/d THC dose was selected based on prior pharmacokinetic studies showing peak plasma THC concentrations in adult mice comparable to those observed in humans after smoking a single cannabis cigarette.^17^ On day 14, mice were briefly anesthetized with isoflurane and infected subcutaneously with 550 *N. brasiliensis* L3 larvae, as previously described.^7,9^ THC or vehicle treatment was paused from day 0 to day 4 postinfection, then resumed until collection on day 8 postinfection. Fecal egg burden was measured at day 7 postinfection, and blood, bronchoalveolar lavage fluid (BALF), lungs, spleens, and small intestines were collected at day 8 postinfection for immune analyses and adult worm counts.

### 2.2. BALF, lung, and blood leukocyte isolation and flow cytometry

BALF, lung, and blood leukocytes were isolated for flow cytometry. BALF was collected by 3 lavages with 0.7 mL sterile 1× PBS. Lungs were dissociated using the mouse Lung Dissociation Kit and gentleMACS Octo Dissociator with Heaters (Miltenyi Biotec). Blood was collected into 4% sodium citrate, diluted, and separated on Histopaque-1077. Viable leukocytes were counted, blocked with CD16/32 and rat IgG, stained with the antibody panels listed in Tables S1 and S2, and analyzed using NovoExpress and FlowJo.

### 2.3. Ex vivo splenocyte stimulation

Spleens were mechanically dissociated, red blood cells were lysed, and splenocytes were cultured in complete T cell culture media. Cells were plated at 5 × 10^6^ cells per well and stimulated with anti-CD3, anti-CD28, and recombinant mouse IL-2 for 72 h at 37 °C. Culture supernatants were collected for cytokine analysis. IFNγ was measured by LEGENDplex Mouse Th1/Th2 Panel, and TNFα was measured by ELISA.

### 2.4. Lung macrophage and eosinophil isolation for bulk RNA sequencing

Whole-lung single-cell suspensions were filtered and enriched by magnetic bead purification. CD11c^+^ cells were isolated using biotinylated anti-mouse CD11c antibody and Streptavidin MicroBeads, and the CD11c-negative flow-through was used to enrich Siglec-F^+^ eosinophils. Enriched cell fractions were lysed in Buffer RLT, and RNA was isolated using the RNeasy Mini Kit. Libraries were prepared and sequenced by the Novogene Corporation. Raw sequencing reads were assessed for quality using FastQC^18^ and summarized with MultiQC.^19^ Adapter sequences and low-quality bases were trimmed using Trimmomatic.^20^ Trimmed reads were aligned to the mouse reference genome using STAR aligner,^21^ and gene-level read counts were generated using FeatureCounts.^22^ Downstream analyses were performed in R. Differential gene expression analysis was conducted using DESeq2.^23^ Genes with an adjusted *P*-value, using Benjamini–Hochberg correction, ≤0.05 and an absolute log2 fold change > 1 were considered differentially expressed unless otherwise indicated. For targeted antigen presentation and costimulation-associated genes, an adjusted *P*-value ≤ 0.05 and absolute log2 fold change > 0.25 were used. Variance-stabilized expression values generated by DESeq2 were used for heatmap visualization. Gene set enrichment analysis was performed using Hallmark gene sets.^24,25^

### 2.5. Principal component analysis

For Principal component analysis (PCA), genes with at least 10 counts in at least 2 samples were variance-stabilized using DESeq2. PCA was performed on the 500 most variable genes using centered, unscaled expression values and plotted in ggplot2 by treatment, infection status, and cell type.

### 2.6. Digital cell quantification analysis

Immune-cell deconvolution was performed in R using immunedeconv. Mouse Ensembl identifiers were mapped to gene symbols, duplicate symbols were summed, and digital cell quantification (DCQ) was used to estimate relative macrophage and dendritic-cell enrichment. Nonnegative estimates were normalized within each sample and interpreted as inferred relative composition, not direct cell frequency.

### 2.7. Lung immunofluorescent staining and imaging

Lungs were inflated with 4% paraformaldehyde, fixed overnight in 10% neutral buffered formalin, transferred to 70% ethanol, and paraffin-embedded by the UCLA Translational Pathology Core Laboratory. Five-micrometer FFPE sections were stained with antibodies against Collagen VI and GSL I (Table S3), quenched for autofluorescence, mounted with DAPI-containing antifade medium, and imaged at 20× on a Keyence microscope. Collagen VI signal was quantified in Fiji/ImageJ as integrated density per field after 16-bit grayscale conversion and background subtraction.

### 2.8. Statistical analysis

Statistical analyses of non-sequencing data were performed using GraphPad Prism. Fecal egg and adult worm burdens were analyzed using an unpaired *t*-test, as these comparisons involved 2 independent treatment groups. Outcomes measured across both treatment and infection conditions, including immune-cell frequencies, cytokine concentrations, and Collagen VI staining, were analyzed by 2-way ANOVA to evaluate the main effects of treatment, infection, and their interaction. Tukey's multiple-comparisons test was used for post hoc pairwise comparisons. Differential gene expression was analyzed in R using DESeq2, which models RNA-sequencing count data using a negative binomial distribution. Benjamini–Hochberg correction was applied to control the false-discovery rate. Genes with an adjusted *P*-value < 0.05 and an absolute log_2_ fold change > 1 were considered differentially expressed, except for the targeted antigen-presentation and costimulation analysis, for which an absolute log_2_ fold change > 0.25 was used. Hallmark gene-set enrichment results with an adjusted *P*-value < 0.05 were considered significant. Data are presented as mean ± SEM unless otherwise indicated, and *P* < 0.05 was considered statistically significant.

## 3. Results

### 3.1. THC exposure does not alter parasite burden following *N. brasiliensis* infection but affects circulating leukocyte responses

Cannabis use has been associated with altered helminth burden in a human population,^6^ raising the possibility that cannabinoid exposure may influence host parasite responses. To determine whether THC exposure altered helminth burden and infection-associated immune responses, we employed an established murine model of helminth infection with *N. brasiliensis* (Nb), a rodent hookworm that infects the lung and small intestine with an acute infection cycle of 7 to 10 d.^7,8,26^ Mice were treated intraperitoneally with THC or vehicle for 2 weeks prior to Nb infection, followed by assessment of infection-associated weight change, fecal egg burden, and intestinal worm burden (Fig. 1a). Following Nb infection, vehicle- and THC-treated mice showed acute weight loss between days 2 and 4 postinfection, consistent with the timing of Nb-associated lung migration and tissue injury (Fig. 1b). However, THC treatment did not significantly alter fecal egg burden or adult worm burden compared with vehicle-treated infected controls (Fig. 1c and d).

**Figure 1:**
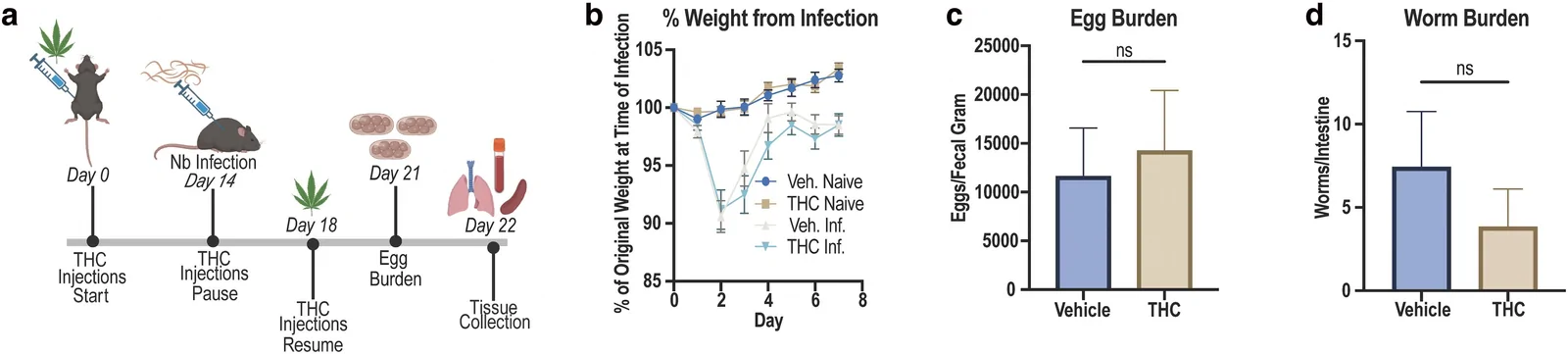
THC exposure does not alter infection-induced weight loss or parasite burden following *Nippostrongylus brasiliensis* infection. Vehicle- and THC-treated mice were left naïve or infected with Nb. a) Experimental design. Created by Biorender. b) Percent body weight change. c) Fecal egg burden. d) Adult worm burden. Data are mean ± SEM pooled from 2 independent experiments (*n* = 4 to 7 mice per group). Statistical analysis was performed using an unpaired *t*-test for egg burden and worm burden. ns, not significant.

We next examined whether THC altered immune cell responses following Nb infection. Analysis was conducted 8 d postinfection, consistent with prior work from our lab demonstrating immune changes influenced by cannabinoid receptor signaling.^5,10^ Whole blood flow cytometry was performed to quantify monocytes, eosinophils, and CD4^+^ T cells (Figure S1). Nb infection increased circulating eosinophil frequencies in vehicle-treated mice, whereas this infection-induced eosinophil response was reduced in THC-treated animals (Fig. 2a). Nb infection also increased circulating monocyte frequencies in vehicle-treated mice, and THC-treated infected mice had reduced circulating monocyte frequencies compared with vehicle-treated infected controls (Fig. 2b). In contrast, CD4^+^CD25^+^ regulatory T cell frequencies were increased in THC-treated infected mice (Fig. 2c). Total CD4+ T cell frequencies in the blood were not significantly altered by THC treatment (Figure S1), indicating that THC did not broadly impact the proportion of circulating CD4+ T cells.

**Figure 2:**
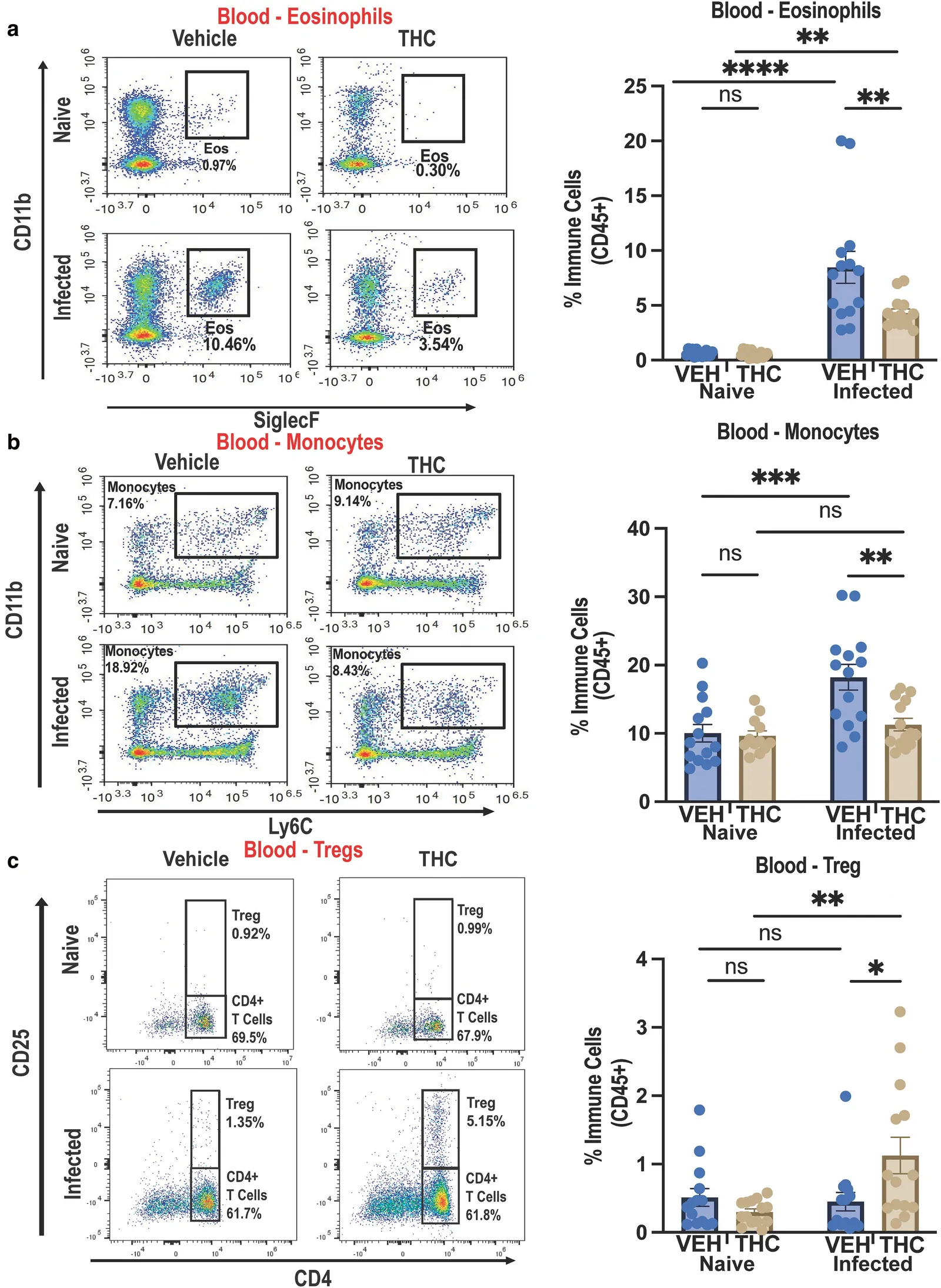
THC exposure reduces infection-induced circulating eosinophilia and alters circulating leukocyte responses. Whole-blood leukocytes were assessed in vehicle- and THC-treated naïve and Nb-infected mice. a to c) Representative plots and frequencies of eosinophils (a), monocytes (b), and CD4^+^CD25^+^ regulatory T cells (c) among CD45^+^ cells. Data are mean ± SEM pooled from 2 independent experiments (*n* = 4 to 7 mice per group). Two-way ANOVA with Tukey's multiple-comparisons test. *P < 0.05; **P < 0.01; ***P < 0.001; ****P < 0.0,001; ns, not significant.

Together, these data indicate that THC exposure does not significantly alter parasite burden or infection-associated weight loss following Nb infection but does alter systemic immune responses. Specifically, THC limited infection-induced circulating eosinophilia and monocyte responses while increasing regulatory T cell frequencies. Overall, these findings support a model in which THC does not broadly suppress anti-helminth immunity but instead shifts the balance of systemic inflammatory and regulatory immune responses during type 2 inflammation.

### 3.2. THC and helminth infection induce broad transcriptional changes in lung CD11c^+^ enriched macrophages and eosinophils

We next evaluated lung-specific changes. Although THC reduced circulating eosinophils and monocytes, BAL and lung eosinophil and myeloid cell frequencies in naïve mice or following Nb infection were not significantly changed with THC exposure (Figures S2 and S3). We investigated whether THC altered the gene expression profiles of these innate immune cells within the lungs of naïve and Nb-infected mice that had been exposed to vehicle or THC (Fig. 3a). Lung single-cell suspensions were processed for column purification to isolate CD11c^+^ alveolar macrophages (86% purity). In the infected mouse groups, which had sufficient eosinophil numbers, the flow-through was next used for Siglec-F column purification to isolate eosinophils (87% purity). Following bulk RNA sequencing and standard quality control filtering, PCA revealed distinct clustering according to cell type and infection status (Figure S4a, *n* = 4 per group). Additional validation analysis was conducted to determine macrophage purity of the CD11c^+^ fraction by DCQ, which predicted that macrophages were the dominant cell subset in all groups investigated with minimal dendritic cell contamination (Figure S4b). We also evaluated cannabinoid receptor expression in the enriched macrophages and eosinophils and observed higher CB2R mRNA in all groups compared to CB1R, but no changes in CB1R or CB2R expression with THC exposure or infection (Figure S5a). We assessed the overall transcriptional impact of infection and THC exposure by comparing the number of differentially expressed genes across RNA sequencing datasets. In CD11c^+^ lung macrophage-enriched cells, Nb infection induced the greatest transcriptional changes in both vehicle- and THC-treated mice; vehicle-treated macrophage-enriched cells had 2,956 genes upregulated and 2,980 genes downregulated with infection, compared to THC-treated macrophage-enriched cells (3,045 genes up versus 3,502 genes down with infection) (Fig. 3b). While THC did not substantially affect macrophage gene expression from naïve mice (254 genes up versus 184 genes down with THC), there were more genes that were changed in the infected mouse groups (Fig. 3c). Eosinophils had more downregulated (1,546) than upregulated genes (688), followed by macrophages with 1,136 genes downregulated versus 925 genes upregulated with THC exposure in the infected groups. Together, these data indicate that THC has a broad transcriptional impact on macrophages and eosinophils during infection with a greater effect in suppressing gene expression.

**Figure 3:**
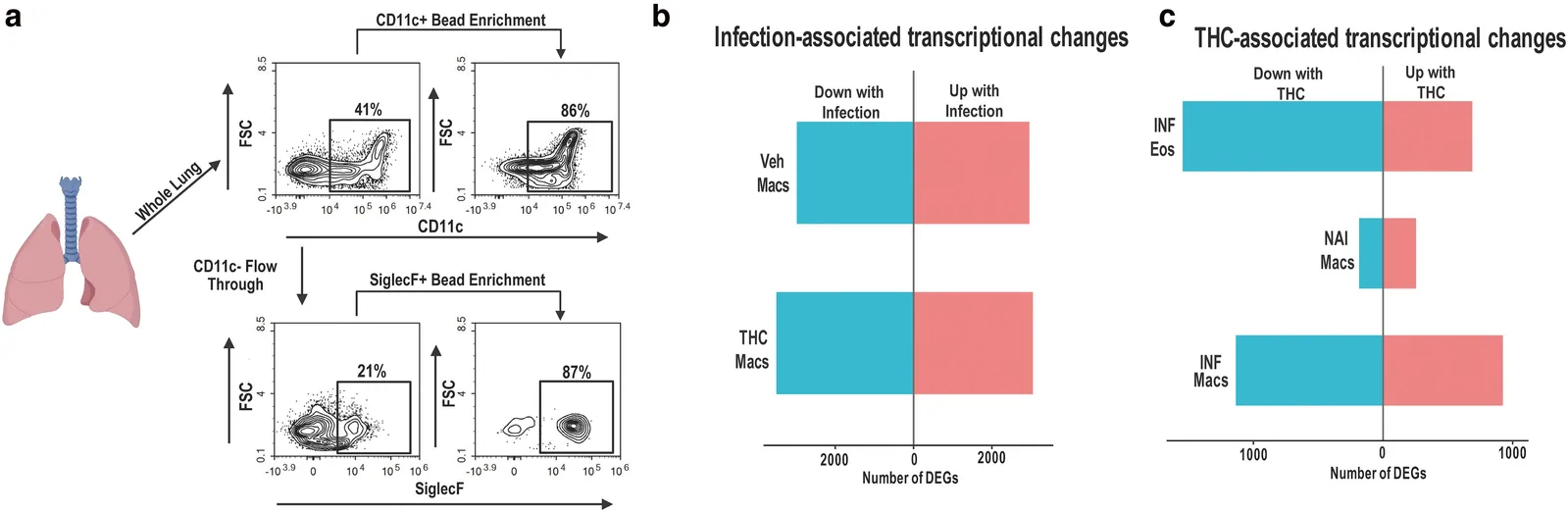
THC exposure and helminth infection induce broad transcriptional changes in lung macrophage-enriched CD11c^+^ cells and eosinophils. Vehicle- and THC-treated mice were left naïve or infected with Nb. a) Magnetic sorting strategy for CD11c^+^ macrophage-enriched cells and CD11c^−^Siglec-F^+^ eosinophils. b) Differentially expressed gene counts comparing naïve and infected CD11c^+^ macrophage-enriched cells. c) Differentially expressed gene counts after THC exposure in infected eosinophils, naïve macrophage-enriched cells, and infected macrophage-enriched cells. Differential expression was defined by DESeq2 adjusted *P* < 0.05 and absolute log_2_ fold change > 1. Data are from one experiment with 7 mice per group pooled into 4 RNA-sequencing libraries per group.

Given prior evidence that THC can alter macrophage inflammatory potential,^12–14^ we examined the genes and functional pathways that were changed in macrophages from naïve mice upon THC exposure (Fig. 4, Table 1). Transcripts reduced in THC-treated naïve cells included chemokine and inflammatory signaling-associated genes such as *Cxcl1*, *Cxcl2*, *Il2rg*, *Cish*, and *Scimp*, as well as heat shock-associated genes including *Hsph1* and *Hspa8*.^27–32^ In contrast, genes increased with THC included *Cirbp*, *Cd300lf*, *Adap2*, *Rgcc*, *Pex11a*, *Cerk*, and *Pla2g6*, consistent with altered stress adaptation, inhibitory signaling, and lipid handling.^33–35^ Gene set enrichment analysis supported this basal shift in CD11c^+^ lung macrophage-enriched cells (Fig. 4b). THC-treated naïve cells showed enrichment of pathways associated with TGFβ signaling, heme metabolism, and fatty acid metabolism. In contrast, vehicle-treated cells were enriched for inflammatory response, TNFα signaling via NFκB, IL-6/JAK/STAT3 signaling, interferon responses, complement, and allograft rejection pathways.

**Figure 4:**
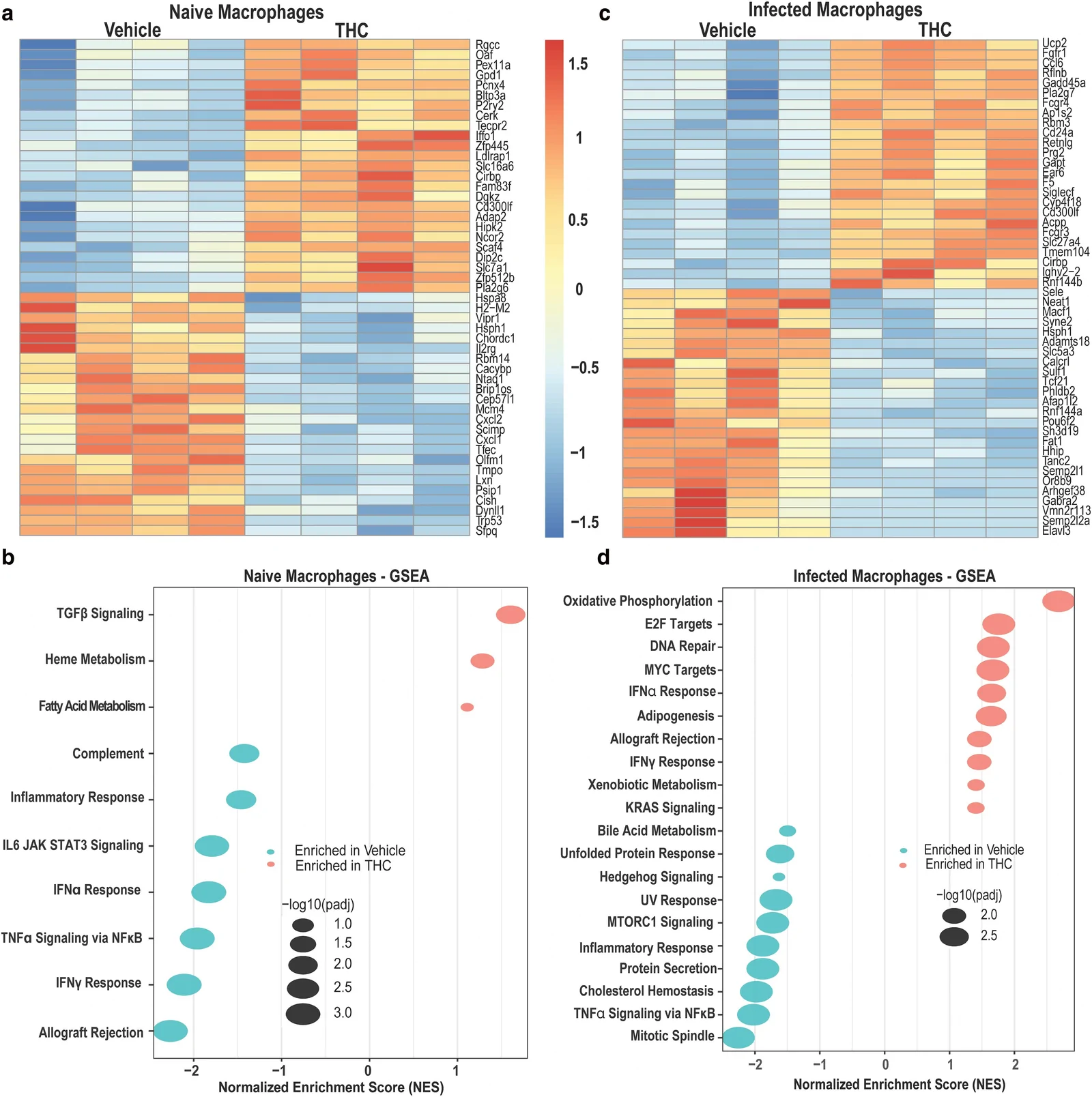
THC dampens basal and Nb-induced inflammatory and tissue-remodeling programs in CD11c^+^ lung macrophage-enriched cells. CD11c^+^ macrophage-enriched cells from naïve or Nb-infected mice treated with vehicle or THC were analyzed by bulk RNA sequencing. a, c) Heatmaps of the top 50 differentially expressed genes in naïve (a) and Nb-infected (c) comparisons. b, d) Hallmark GSEA for naïve (b) and Nb-infected (d) comparisons. Heatmap genes met adjusted *P* < 0.05 and absolute log_2_ fold change > 1; GSEA pathways met adjusted *P* < 0.05. Data are from one experiment with 7 mice per group pooled into 4 RNA-sequencing libraries per group.

**Table 1:** Naïve lung CD11c^+^ enriched macrophage differentially expressed genes.

Category	Down in THC	Up in THC	Interpretation
**Stress response**	*Hsph1*, *Hspa8*, *Chordc1*	*Cirbp*	THC reduces classical heat-shock stress, enhances stress buffering
**Chemokines**	*Cxcl1*, *Cxcl2*	—	Vehicle macs show higher basal chemokine production
**Cytokine signaling and activation**	*Il2rg*, *Cish*, *Tfec*, *Scimp*	*Cd300lf*, *Adap2* (inhibitory)	THC upregulates inhibitory receptors, lowering activation potential
**Cell cycle/proliferation**	*Mcm4*, *Tmpo*, *Psip1*	*Rgcc*, *Pex11a*, *Gpd1*, *Slc16a6*	THC shifts toward metabolic/FAO genes rather than proliferation
**Lipid turnover**	—	*Cerk*, *Pla2g6*	THC enhances lipid-handling without inflammatory activation

We next examined whether THC altered CD11c^+^ lung macrophage-enriched cell responses during Nb infection. Differential gene expression analysis showed that THC altered the transcriptional response of infected CD11c^+^ lung macrophage-enriched cells (Fig. 4c, Table 2). THC reduced expression of genes associated with pathogen sensing, chemotaxis, motility, and remodeling. Reduced transcripts included *Tlr4*, *Fpr1*, and *P2ry13*, as well as heat shock-associated genes such as *Hsph1*, *Hsp90aa1*, and *Hspa8*.^27,36–39^ THC also reduced genes linked to cytoskeletal remodeling and fibrotic programs, including *Myo1f*, *Bin2*, *Spice1*, and *P4ha1*.^40–43^ Conversely, THC increased expression of genes associated with stress adaptation, regulatory signaling, and metabolic remodeling, including *Cirbp*, *Rbm3*, *Foxred2*, *Eci2*, *Pik3ip1*, *Lpxn*, *Susd3*, *Retnlg*, and *Pdcd4*.^33,44–47^

**Table 2:** Infected lung CD11c^+^-enriched macrophage differentially expressed genes.

Category	Down in THC	Up in THC	Interpretation
**Pathogen sensing/chemotaxis**	*Tlr4*, *Fpr1*, *P2ry13*	—	THC reduces PRR/chemotaxis signaling
**Stress response**	*Hsph1*, *Hsp90aa1*, *Hspa8* (heat-shock proteins)	*Cirbp*, *Rbm3*, *Foxred2* (cold-shock/stress-buffering)	THC shifts from inflammatory stress responses to protective stress buffering
**Metabolism**	*Pla2g15* (lipid metabolism for inflammation)	*Eci2* (FAO)	THC redirects metabolism away from inflammatory lipid use toward FAO
**Cytoskeletal/adhesion**	*Myo1f*, *Bin2*, *Spice1*	—	Reduced motility/adhesion signatures
**Fibrosis/ECM remodeling**	*P4ha1*	—	Lower fibrosis/ECM remodeling potential
**Immune regulation**	—	*Pik3ip1*, *Lpxn*, *Susd3*, *Retnlg*, *Pdcd4*	THC increases inhibitory/checkpoint signals

Pathway analysis confirmed that THC altered macrophage-enriched transcriptional responses during infection. In vehicle-treated macrophages from infected mice, pathways associated with inflammatory response, TNFα signaling via NFκB, mTORC1 signaling, protein secretion, unfolded protein response, cholesterol homeostasis, and mitotic spindle were enriched. On the other hand, THC exposure promoted the enrichment of genes associated with oxidative phosphorylation, E2F targets, DNA repair, Myc targets, interferon responses, adipogenesis, and xenobiotic metabolism pathways (Fig. 4d). Together, these macrophage-enriched datasets from the lungs of naïve and Nb-infected mice indicate that THC reduces inflammatory, chemokine-associated, and remodeling programs, especially with infection, and instead promotes genes associated with metabolic adaptation, stress regulation, and immune restraint.

Although THC reduced circulating eosinophilia, lung eosinophil accumulation following Nb infection was not significantly reduced with THC exposure (Figure S2). We therefore investigated whether THC altered the transcriptional state of eosinophils that reached the lung during infection. Differential gene expression analysis revealed that THC altered the transcriptional profile of infected lung eosinophils (Fig. 5a, Table 3). Among the top 50 differentially expressed genes, THC-treated eosinophils showed increased expression of transcripts associated with stress adaptation and cellular buffering, including *Ucp2*, *Cirbp*, *Rbm3*, and *Gadd45a*.^33,44,48–50^ THC-treated eosinophils also showed increased expression of genes associated with lipid mediator regulation and inhibitory receptor signaling, including *Pla2g7*, *Cyp4f18*, *Cd300lf*, and *Siglecf*.^34,51–54^ In contrast, transcripts associated with adhesion, migration, and inflammatory stress, including *Macf1*, *Syne2*, *Afap1l2*, *Neat1*, and *Hsph1*, were reduced in THC-treated eosinophils.^27,55,56^

**Figure 5:**
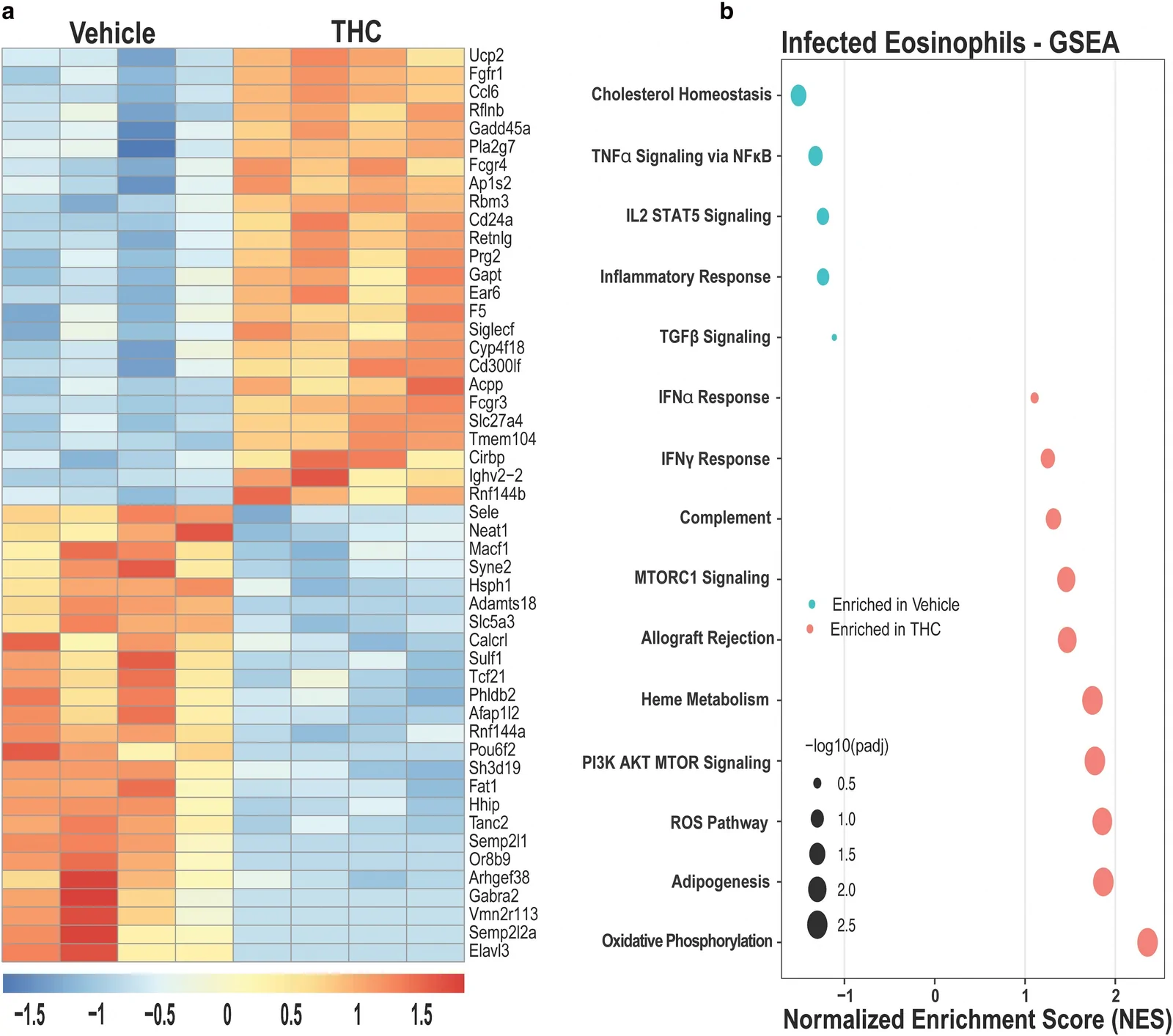
THC shifts infected lung eosinophils from inflammatory NFκB/STAT signaling to mTOR signaling and oxidative phosphorylation. CD11c^−^Siglec-F^+^ lung eosinophils from vehicle- and THC-treated Nb-infected mice were analyzed by bulk RNA sequencing. a) Heatmap of the top 50 differentially expressed genes. b) Hallmark GSEA. Heatmap genes met adjusted *P* < 0.05 and absolute log_2_ fold change > 1; GSEA pathways met adjusted *P* < 0.05. Data are from one experiment with 7 mice per group pooled into 4 RNA-sequencing libraries per group.

**Table 3:** CD11c-Siglec-F+ enriched lung eosinophil differentially expressed genes.

Category	Down in THC	Up in THC	Interpretation
**Stress response/antioxidant**	—	*Ucp2*, *Cirbp*, *Rbm3*, *Gadd45a*	Enhanced buffering of oxidative and cellular stress
**Lipid mediator metabolism**	—	*Pla2g7*, *Cyp4f18*	Reduced PAF/LTB4 signaling capacity (neutrophil recruitment)
**Inhibitory receptors**	—	*Cd300lf*, *Siglecf*	Higher activation threshold
**Cytoskeleton/adhesion**	*Macf1*, *Syne2*, *Afap1l2*	—	Lower migratory and adhesion potential
**Stress/inflammatory regulators**	*Neat1*, *Hsph1*	—	Reduced stress-induced inflammatory signaling

Gene set enrichment analysis further supported a THC-induced shift in the eosinophil metabolic state. Pathways associated with oxidative phosphorylation, adipogenesis, reactive oxygen species, PI3K–AKT–mTOR signaling, and heme metabolism were enriched in THC-treated eosinophils (Fig. 5b). In contrast, inflammatory response, TNFα signaling through NFκB, IL-2 STAT5 signaling, TGFβ signaling, and cholesterol homeostasis pathways were enriched in vehicle-treated eosinophils. We investigated if THC treatment affected expression of type 2 cytokine/chemokine and their receptors associated with eosinophil recruitment, including eotaxin/CCR3 and IL-5/IL-13 receptor signaling (Figure S5). THC treatment significantly increased CCR3 expression in both macrophages and eosinophils of helminth-infected mice, but there were no significant differences in eotaxin expression. Together, these data suggest that THC does not limit eosinophil accumulation in the lung during Nb infection but instead alters the transcriptional state of lung eosinophils that enter the tissue. Here, THC may restrain eosinophil inflammatory potential at 2 levels: by reducing the circulating eosinophil response and by shifting infected lung eosinophils toward a less inflammatory, more antioxidant-associated transcriptional state after tissue entry.

### 3.3. THC alters antigen presentation and costimulatory gene expression while limiting inflammatory T cell activation

Given that THC altered circulating leukocyte responses and triggered anti-inflammatory transcriptional programs in CD11c^+^ lung macrophages, we hypothesized that inflammatory T cell responses may be changed. Flow cytometric analysis of BALF showed that THC-treated infected mice had increased CD4^+^ T cell frequencies compared with vehicle-treated infected mice (Fig. 6a and b). To determine whether this increase corresponded with enhanced inflammatory T cell function, splenocytes from vehicle- and THC-treated mice were stimulated ex vivo with anti-CD3/CD28 and IL-2 for 72 h, and cytokine secretion was measured in culture supernatants (Fig. 6c). Splenocytes from THC-treated infected mice generated significantly less IFNγ and TNFα following stimulation compared with splenocytes from vehicle-treated infected controls (Fig. 6d and e), although there were no differences in IL-4 and IL-5 production (Figure S5). These data indicate that THC increased CD4^+^ T cell accumulation in the BALF; however, its systemic impact was immunosuppressive, characterized by increased blood Tregs and reduced inflammatory cytokine production by splenic T cells.

**Figure 6:**
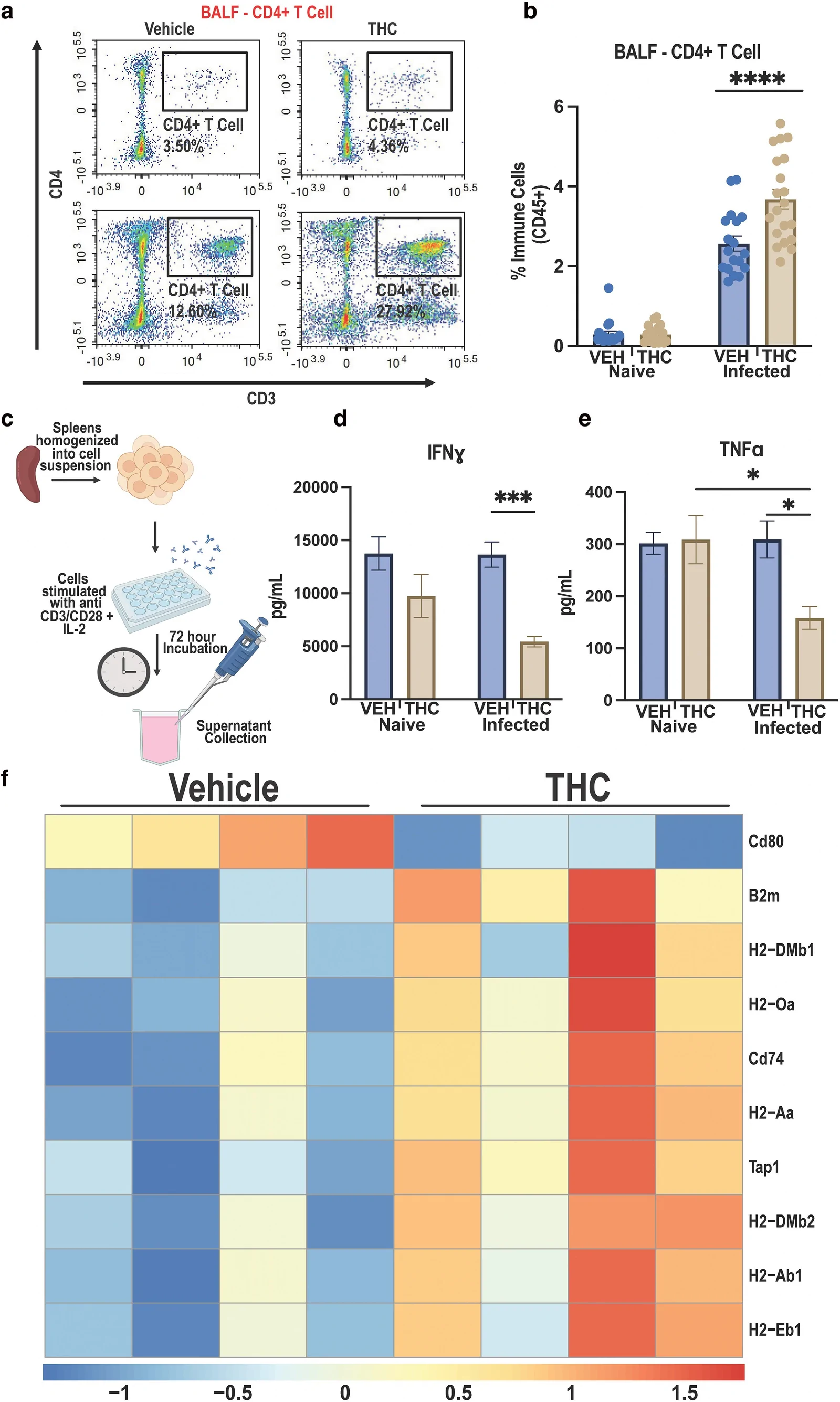
THC limits inflammatory T cell activation while preserving antigen presentation programs. a, b) Representative BALF flow plots (a) and CD4^+^ T cell frequencies among CD45^+^ cells (b). c) Ex vivo splenocyte stimulation schematic. Created with Biorender. d, e) IFNγ and TNFα concentrations after stimulation. f) Targeted heatmap of MHC class II, antigen-presentation, and costimulation genes in infected CD11c^+^ macrophage-enriched cells. Flow cytometry and cytokine data are mean ± SEM pooled from 2 independent experiments (*n* = 4 to 7 mice per group). RNA-Seq data are from one experiment with 7 mice per group pooled into 4 libraries. Two-way ANOVA with Tukey's test; genes in (f) met adjusted *P* < 0.05 and absolute log_2_ fold change > 0.25. *P < 0.05; ***P < 0.001; ****P < 0.0001.

Given that antigen-presenting cells regulate T cell activation through MHC-dependent antigen presentation and CD80/CD86-mediated costimulatory signaling,^57^ we next examined antigen presentation and costimulation-associated genes in CD11c^+^ lung macrophages. Under naïve conditions, there were no significant differences in antigen presentation or costimulation-associated genes between vehicle- and THC-treated mice. During infection, however, THC-treated CD11c^+^ lung macrophages maintained or increased expression of multiple MHC class II and antigen presentation-associated genes, including *H2-Aa*, *H2-Ab1*, *H2-Eb1*, *Cd74*, *H2-DMb1*, *H2-DMb2*, *H2-Oa*, *B2m*, and *Tap1*^58^ (Fig. 6f). We validated increased surface expression of MHC class II in THC-treated helminth-infected mice by flow cytometry of the lung CD11c^+^ cells (Figure S2b). In contrast, *Cd80* expression was reduced in THC-treated infected cells, while *Cd86* was not significantly changed. Together, these data indicate that THC-treated mice have increased airway CD4^+^ T cell frequencies but reduced inflammatory cytokine output following stimulation. This pattern suggests that THC does not block antigen presentation or fully eliminate costimulatory potential but may partially dampen costimulatory signaling in a manner that limits inflammatory T cell activation.^12^

### 3.4. THC treatment is associated with retained lung Collagen VI following Nb infection

Based on the transcriptomic profiling results showing that THC altered inflammatory and remodeling-associated transcriptional programs in lung macrophage-enriched cells, we next assessed whether THC was associated with tissue-level changes in the lung. We chose to investigate collagen deposition in the lung as a measure of tissue remodeling following the severe infection-induced injury caused by Nb burrowing through the lung during the acute infection stages. Immunofluorescent staining for Collagen VI was performed on lung sections from vehicle- and THC-treated naïve and infected mice (Fig. 7a). Vehicle-treated infected mice showed reduced Collagen VI staining compared with vehicle naïve controls, consistent with infection-associated disruption or remodeling of the lung extracellular matrix.^9,16,26^ In contrast, Collagen VI signal was not significantly reduced in THC-treated infected mice compared with THC-treated naïve controls (Fig. 7b). Together, these data suggest that Nb infection is associated with loss of lung Collagen VI in vehicle-treated mice, whereas THC-treated mice retain Collagen VI following infection. This pattern suggests that THC-associated immune reprogramming is accompanied by altered extracellular matrix remodeling during Nb-induced lung inflammation.^59^

**Figure 7:**
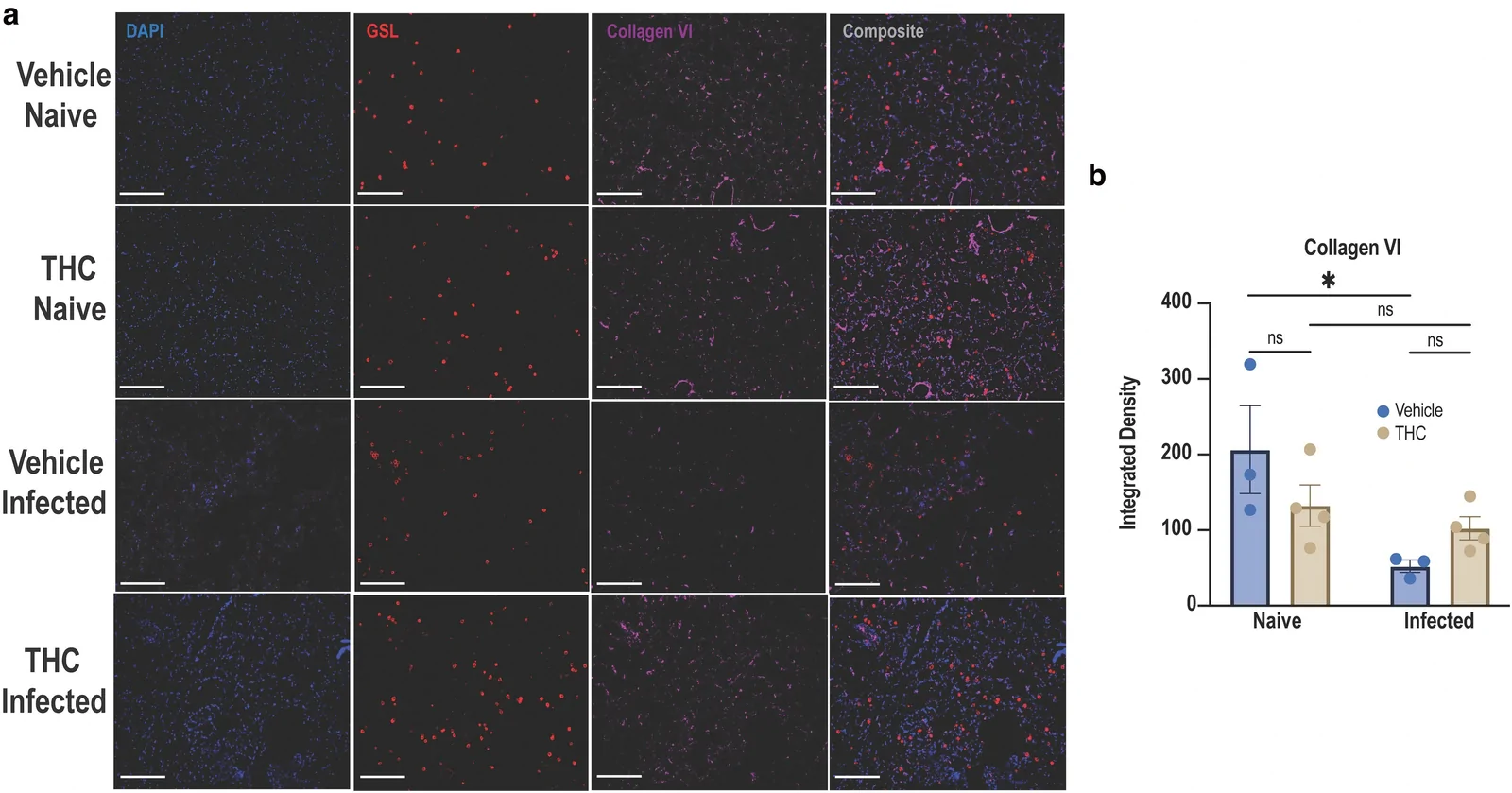
THC treatment is associated with retained lung Collagen VI following Nb infection. Lung extracellular matrix remodeling was assessed in vehicle- and THC-treated naïve and Nb-infected mice. a) Representative DAPI, GSL I, and Collagen VI immunofluorescence images. Scale bar = 20 µm. b) Quantification of Collagen VI integrated density. Data are from individual animals with mean ± SEM. Two-way ANOVA with Tukey's multiple-comparisons test. *P < 0.05; ns, not significant.

## 4. Discussion

The goal of this study was to determine how THC exposure alters immune responses during type 2 inflammation. To achieve a controlled experimental environment, we employed the *N. brasiliensis* infection model and investigated whether THC impacted parasite burden, systemic immune activation, T cell inflammatory function, and lung eosinophil and macrophage gene expression. We found that THC did not alter infection-associated weight loss or parasite burden. Instead, THC restrained infection-induced eosinophilia, increased regulatory T cells, and reduced T cell-derived inflammatory cytokines. Bulk RNA sequencing of lung innate immune cells further showed that THC reduced basal and infection-associated inflammatory programming in CD11c^+^ macrophage-enriched cells and shifted infected lung eosinophils away from migratory and inflammatory activation to an altered metabolic state involving mTOR signaling and oxidative phosphorylation.

An important finding from this study is that THC exposure did not alter *N. brasiliensis* burden. This is notable because human studies have suggested a relationship between cannabis use and helminthiasis. Among Aka foragers of the Congo Basin, urinary THCA levels were negatively associated with parasite infection and reinfection, supporting the possibility that cannabis exposure may influence helminth burden.^6^ However, that study evaluated whole cannabis use in a human population, where additional cannabinoids, route of exposure, tobacco co-use, nutrition, behavior, and environmental variables may contribute to parasite outcomes. In our controlled preclinical model, THC exposure alone did not alter egg or worm burden. These findings suggest that purified THC exposure is not sufficient to reproduce the cannabis-associated reduction in parasite burden observed in human populations. Instead, the major effects of THC in this study were immunologic rather than antiparasitic.

Although parasite burden was unchanged, THC exposure altered the host immune response. The reduction in infection-induced circulating eosinophilia is consistent with prior work implicating cannabinoid signaling in eosinophilic inflammation during helminth infection. Inhibition of peripheral cannabinoid receptor subtype 1 during *N. brasiliensis* infection increased airway eosinophilia and worsened pulmonary pathology, suggesting that cannabinoid signaling can restrain eosinophilic lung inflammation.^5,10^ Our results indicating reduced circulating eosinophils and decreased inflammatory pathways in lung eosinophils in THC-treated mice are consistent with the broader concept that cannabinoid pathways dampen eosinophil-associated inflammation. RNA sequencing of infected lung eosinophils showed that THC altered eosinophil transcriptional states without reducing lung eosinophil accumulation. THC-treated eosinophils showed increased expression of genes associated with stress adaptation, lipid mediator regulation, and inhibitory receptor signaling, including *Ucp2*, *Cirbp*, *Rbm3*, *Pla2g7*, *Cyp4f18*, *Cd300lf*, and *Siglecf*. Increased *Rbm3* is consistent with prior work linking it to restrained type 2 lung inflammation, while *Cirbp* and *Gadd45a* are defined as stress-response genes with context-dependent roles in inflammation.^33,44,50^ Increased *Cyp4f18*, *Pla2g7*, *Cd300lf*, and *Siglecf* is consistent with altered lipid mediator and inhibitory receptor-associated programs in eosinophils, given prior evidence linking CYP4F18 to leukotriene B4 metabolism and CD300/Siglec-family receptors to eosinophil survival, chemotaxis, and effector function.^34,51–54^ In contrast, genes associated with adhesion, migration, and inflammatory stress, including *Macf1*, *Syne2*, *Afap1l2*, *Neat1*, and *Hsph1*, were reduced. These transcriptional changes suggest that THC does not primarily attenuate eosinophilic inflammation by blocking eosinophil entry into the lung. Instead, THC may alter the activation state of eosinophils that enter inflamed tissue. This distinction is important because eosinophils can contribute to both protective type 2 immunity and tissue pathology depending on their inflammatory context, activation state, and tissue localization.^60^

THC also altered CD11c^+^ lung macrophage-enriched cells before and during infection. Under naïve conditions, THC reduced chemokine and cytokine signaling and heat shock-associated transcripts in CD11c^+^ macrophage-enriched cells, while increasing genes associated with stress buffering, lipid handling, and inhibitory signaling. During infection, THC reduced inflammatory, stress-associated, chemotactic, and remodeling-associated genes, including *Tlr4*, *Fpr1*, *P2ry13*, *Hsph1*, *Hsp90aa1*, and *P4ha1*. Reduced *Tlr4*, *Fpr1*, and *P2ry13* are consistent with dampened pathogen-sensing, formyl peptide receptor, and purinergic inflammatory programs, pathways previously linked to helminth-associated Th2 responses, lung leukocyte recruitment, and macrophage inflammatory regulation.^27,36,37,39,43^ Reduced *P4ha1* is also consistent with decreased collagen-processing or ECM-remodeling-associated transcriptional programs.^43^ These data indicate that THC changes the baseline state of lung macrophage-enriched cells and alters their response during inflammatory challenge. This is consistent with prior studies showing that THC can regulate myeloid cell biology, including inhibition of macrophage costimulatory activity, alveolar macrophage inflammatory responses, and ROS-associated macrophage differentiation.^12–14^ The transcriptional changes observed in macrophage-enriched cells may have implications for tissue remodeling. THC reduced expression of genes associated with motility, inflammatory stress, and fibrotic remodeling, while increasing metabolic and stress-adaptive pathways. Consistent with this transcriptional pattern, vehicle-treated infected mice showed reduced lung Collagen VI staining, whereas THC-treated infected mice retained Collagen VI signal following infection. Collagen VI is a structural extracellular matrix protein, and changes in its deposition may reflect altered tissue remodeling during inflammation. These data suggest that THC-associated immune reprogramming is accompanied by altered lung matrix remodeling.^9,59^

In CD11c^+^ macrophage-enriched cells from infected mice, THC-treated samples maintained or increased expression of multiple MHC class II-associated genes, while *Cd80* was reduced. Because CD80-mediated costimulation contributes to optimal T cell activation and cytokine production, reduced *Cd80* expression may help explain why CD4^+^ T cells accumulated in the airway but showed reduced inflammatory output after stimulation.^57^ The combination of maintained antigen presentation genes, reduced *Cd80*, and reduced TNFα and IFNγ production is consistent with incomplete inflammatory T cell activation or an anergy-like functional state.^12,57,58^ These findings are consistent with prior work showing that THC suppresses proliferation, reduces IFNγ production, and alters Th1/Th2 cytokine balance in activated human T cells.^11^

There remain limitations to this study. First, although THC is a partial cannabinoid receptor agonist, we did not directly test whether the observed effects were mediated through CB1R, CB2R, or cannabinoid receptor-independent pathways.^2,12,14^ Future studies using receptor antagonists, conditional deletion mouse models, or cell-specific approaches will be needed to determine receptor dependence. The route of THC administration is also an important consideration. We used intraperitoneal injection to provide consistent, body-weight-adjusted dosing and to isolate the systemic immunologic effects of THC from potential airway irritation or local pulmonary changes caused by inhaled exposure. However, intraperitoneal administration does not reproduce the pharmacokinetics or local airway exposure associated with human cannabis inhalation, and future studies using vaporized THC will be needed to determine whether the observed immune effects are maintained with a more clinically relevant exposure route. Second, the CD11c^+^ lung population used for RNA sequencing was macrophage-enriched but may also contain dendritic cells or other CD11c-expressing populations. Third, although reduced *Cd80* expression and lower TNFα and IFNγ release are consistent with reduced costimulatory activation,^11,12,57^ direct lung T cell assays will be required to determine whether THC induces T cell anergy, altered proliferation, or changes in antigen-specific effector function. Lastly, many physiological effects of cannabis are mediated by THC; however, pure THC is rarely consumed on its own, as people typically consume whole cannabis or concentrated cannabis extracts. Future studies should evaluate possible differential effects for THC versus whole cannabis, which contains over 100 phytocannabinoid chemicals.^61^

In conclusion, chronic THC exposure reprograms eosinophils and macrophages toward restrained, oxidative, and stress-adaptive phenotypes during type 2 inflammation, associated with reduced inflammatory T cell responses. The immune skewing potential of THC on lung innate immune cells may offer therapeutic potential in inflammatory settings where excessive leukocyte activation contributes to tissue pathology.^9,16,26^

## Supplementary Material

qiag131_Supplementary_Data

## Data Availability

The RNA-sequencing data generated in this study have been deposited in the NCBI Gene Expression Omnibus (GEO) under accession number GSE334156 and can be accessed at https://www.ncbi.nlm.nih.gov/geo/query/acc.cgi?acc=GSE334156 using the reviewer access token krypoeuqtpczhgt.
