# Supplementary material for: Δ9-Tetrahydrocannabinol exposure shifts eosinophil and macrophage transcriptional programs toward an anti-inflammatory phenotype in helminth infection
Source: J Leukoc Biol. 2026 Sep 24;118(10):qiag131. doi: 10.1093/jleuko/qiag131 (PMC13653213; doi:10.1093/jleuko/qiag131)

***Supplemental Table 1*. Whole Lung/Blood Flow Cytometry Panel**

Laser	Fluorophore	Surface	Company	Cat.	Clone
488 nm	FITC	F4/80	Biolegend	123108	BM8
	PerCP, 7AAD	7AAD	Biolegend	420404	N/A
	PerCP Cy5.5	CD4	Biolegend	100540	RM4-5
	PE Cy7	Ly6C	Invitrogen	25-5932-82	HK1.4
637 nm	APC	CX3CR1	Biolegend	149008	SA011F11
	AF700	MHCII	Invitrogen	50-169-17	M5/114.15.2
	APC Cy7	CD11b	Biolegend	101226	M1/70
405 nm	BV510	Ly6G	Biolegend	127627	1A8
	BV570	CD45	Biolegend	103135	30-F11
	BV650	CD86	Biolegend	105036	GL-1
	BV711	CD206	Biolegend	141727	C068C2
561 nm	PE	CD25	Biolegend	113704	SA203G11
	PE Dazzle	SiglecF	Biolegend	155530	S17007L
	PeCy5	CD3ε	eBioscience	15-0031-82	145-2C11
	PE Cy5.5	CD11c	Invitrogen	35-0116-42	3.9

***Supplemental Table 2*. BALF Flow Cytometry Panel**

Laser	Fluorophore	Surface	Company	Cat.	Clone
488 nm	FITC	F4/80	Biolegend	123108	BM8
	PerCP, 7AAD	7AAD	Biolegend	420404	N/A
	PerCP, Cy5.5	CD4	Biolegend	100540	RM4-5
	PerCP eFlour 710	CD45R	eBioscience	41-0452-80	RA3-6B2
	PE Cy7	Ly6C	Invitrogen	25-5932-82	HK1.4
637 nm	APC	CD3	Biolegend	100236	17A2
	AF700	MHCII	Invitrogen	50-169-17	M5/114.15.2
	APC Cy7	CD11b	Biolegend	101226	M1/70
405 nm	BV510	Ly6G	Biolegend	127627	1A8
	BV570	CD45	Biolegend	103135	30-F11
	BV650	CD86	Biolegend	105036	GL-1
	BV711	CD206	Biolegend	141727	C068C2
561 nm	PE Dazzle	Siglec F	Biolegend	155530	S17007L
	PE Cy5.5	CD11c	Invitrogen	35-0116-42	3.9

Supplemental Table 3. **Lung Immunofluorescence Panel**

**Primary**	**Company**	**Cat.**
GSL I Isolectin B4, Biotinylated	Vector Laboratories	B-1205-.5
Rabbit anti-mouse COL6A1 PolyAb	Proteintech	17023-1-AP
**Secondary**	**Company**	**Cat.**
SAV Cy3	Invitrogen	SA1010
Goat anti-Rabbit Cy5	KPL	072-02-15-06

Sup. Fig. 1.

A. PBMC - Eosinophils and Monocyte Gating Strategy

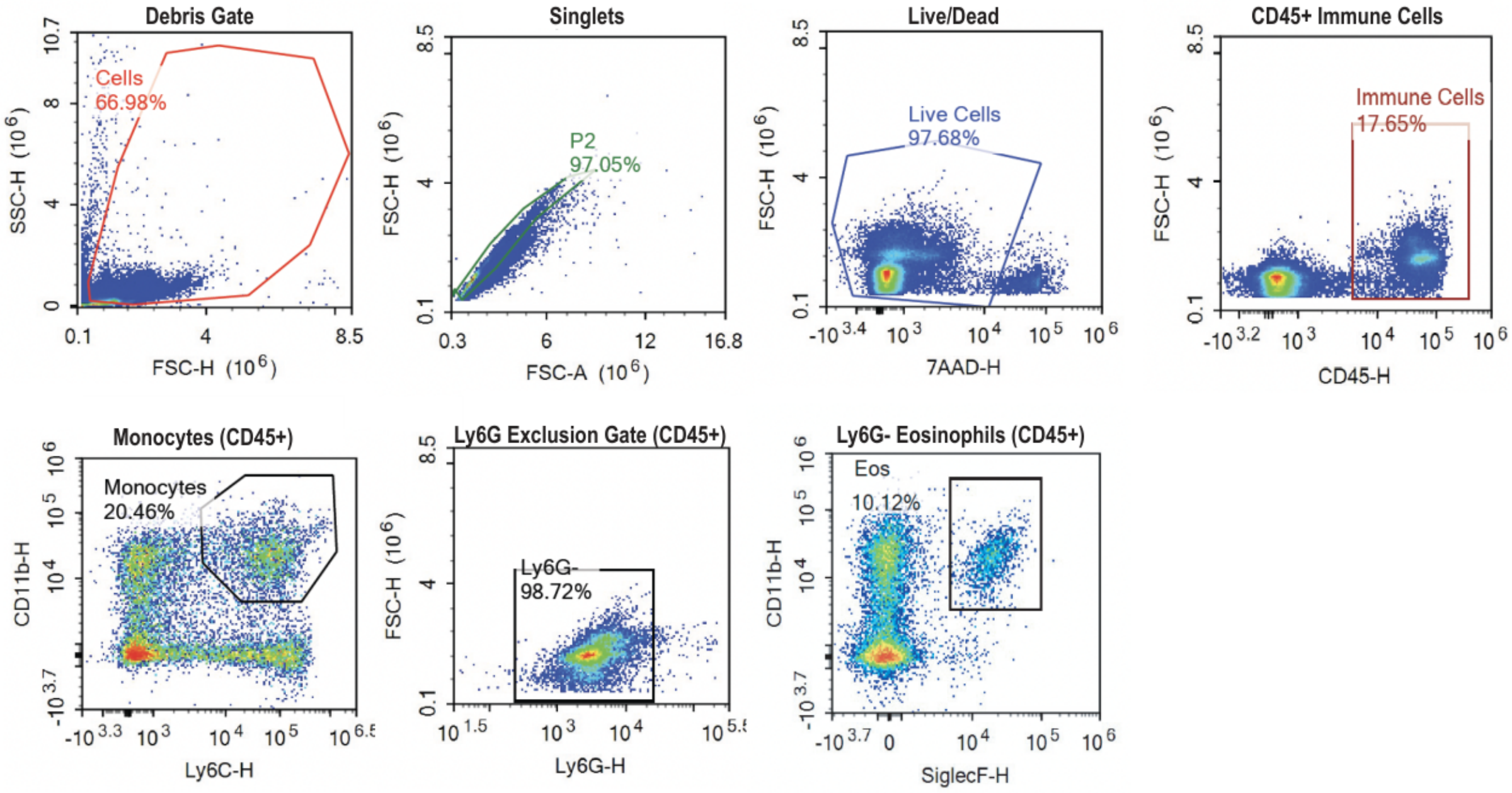

B. PBMC - T Cell Gating Strategy

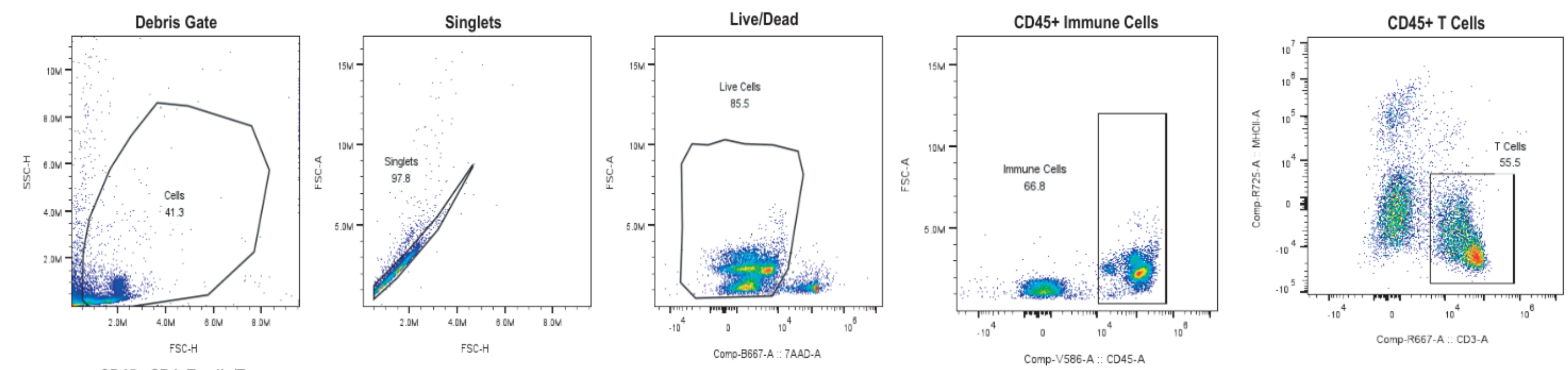

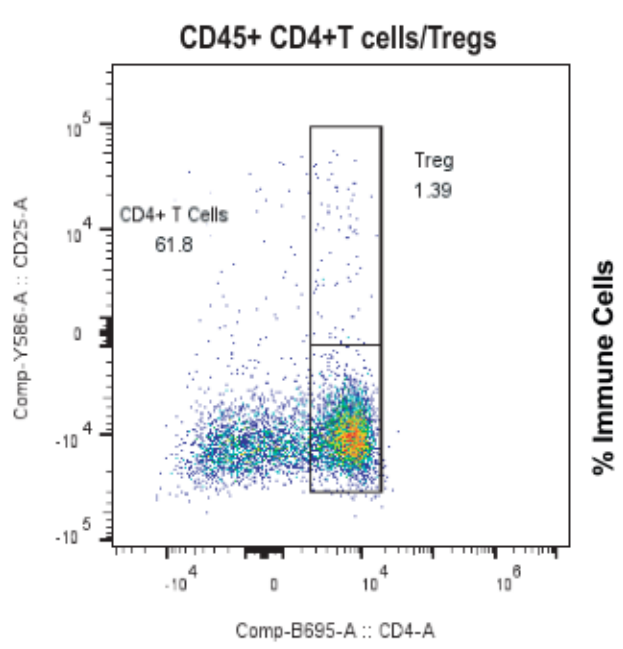

Supplemental Figure 1. Flow cytometry gating strategies for whole blood immune cell analysis. (A) Monocytes and eosinophils gating strategy. (B) $CD4^{+}CD25^{+}$ Tregs gating strategy. (C) $CD4^{+}$ T cell frequency among $CD45^{+}$ cells. Data are mean ± SEM pooled from 2 independent experiments (n=4-7 mice/group/experiment). Analyzed via two-way ANOVA with Tukey's post hoc test. ***p<0.001; ****p<0.0001; ns, not significant.

C.

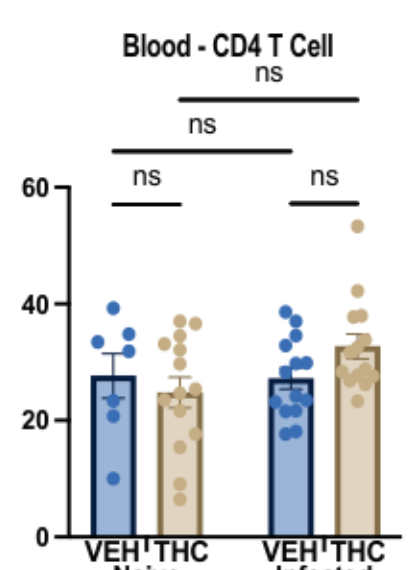

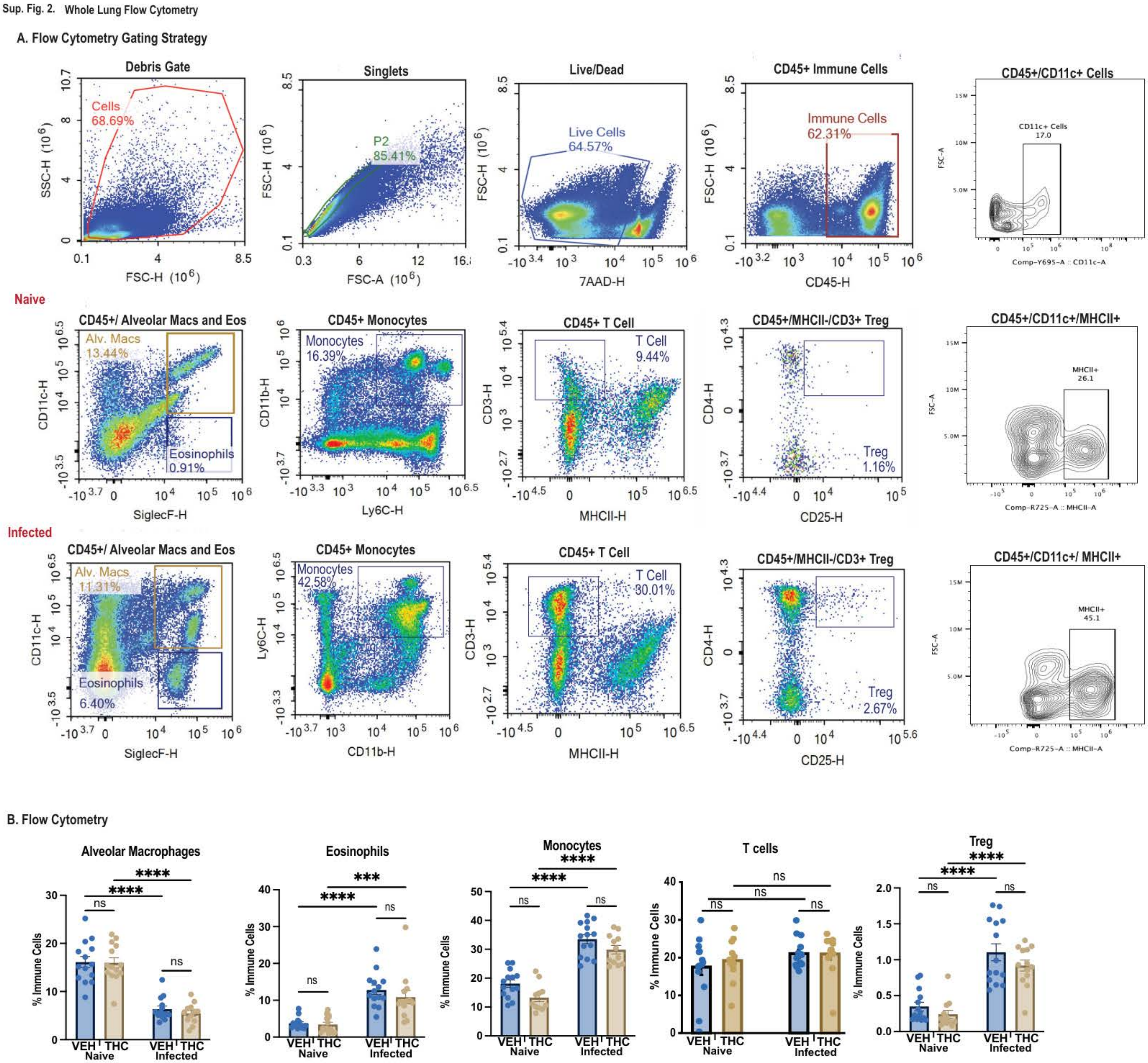

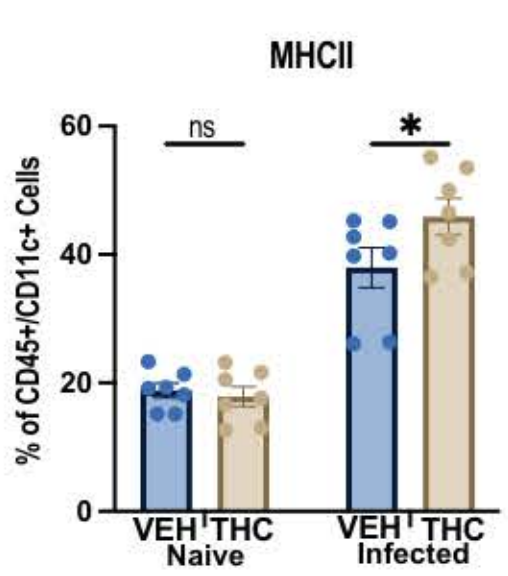

Supplemental Figure 2. Flow cytometry gating strategies and quantification of whole-lung immune cell populations. (A) Naïve lung gating strategy (exclusion of debris, doublets, dead cells; selection of $CD45^+$ cells). Alveolar macrophages and eosinophils were identified by CD11c and Siglec-F. $CD11c^+$ cells were gated from $CD45^+$, followed by $MHCII^+$ quantification in naïve and Nb-infected lungs. (B–D) Cell frequencies: (B) Alveolar macrophages in $CD45^+$, (C) Eosinophils in $CD45^+$, and (D) $MHCII^+$ cells in $CD45^+CD11c^+$. Data: mean ± SEM. (B, C) Pooled from 2 experiments (n=4-7 mice/group/exp; ordinary two-way ANOVA with Tukey's test). (D) Single experiment (n=7 mice/group; full-interaction two-way ANOVA with Šídák's test comparing vehicle vs. THC per infection state). *$p<0.05$; ***$p<0.001$; ****$p<0.0001$; ns, not significant.

Sup. Fig. 3. BALF Flow Cytometry

A. Flow Cytometry Gating Strategy

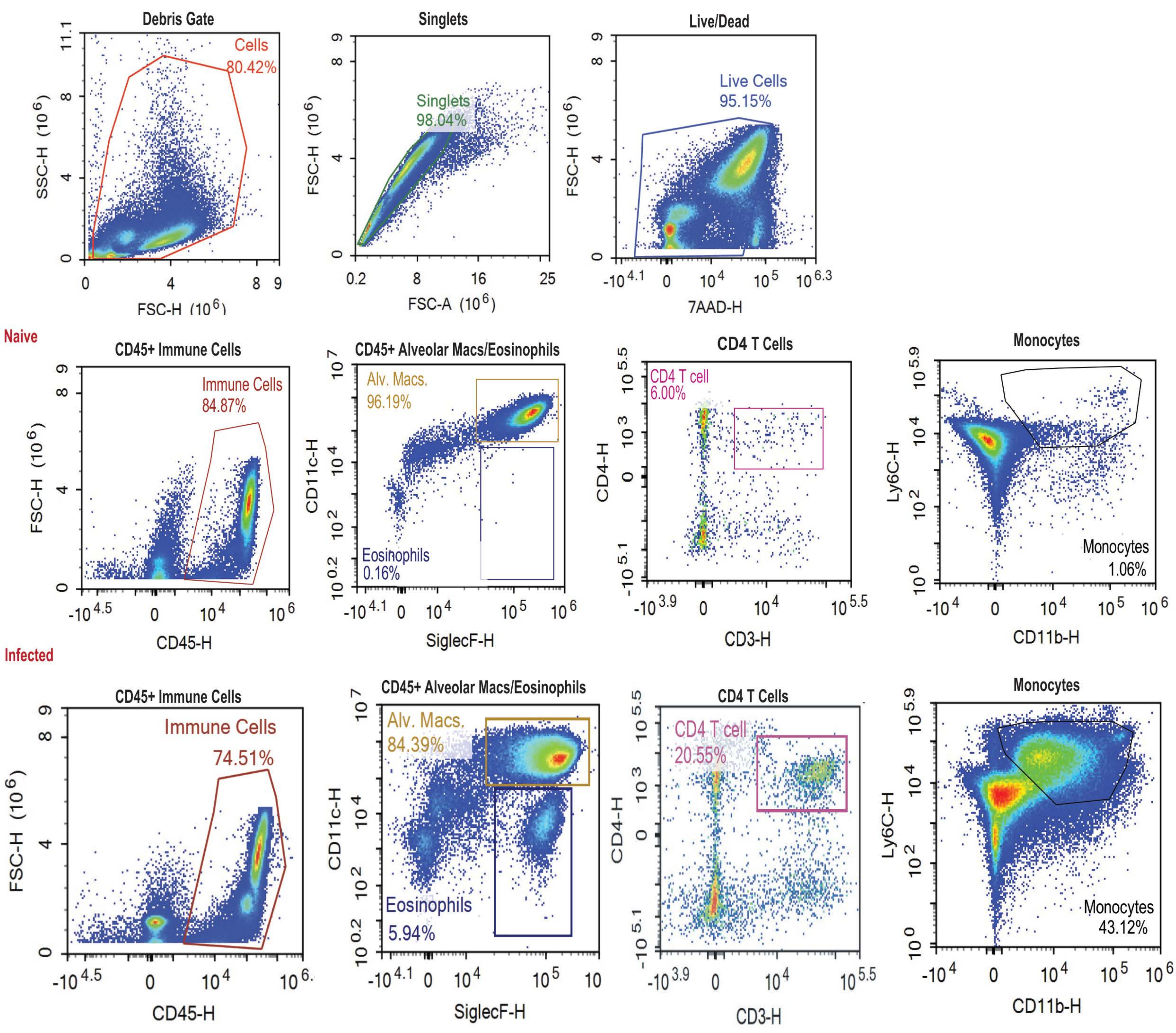

B. Flow Cytometry

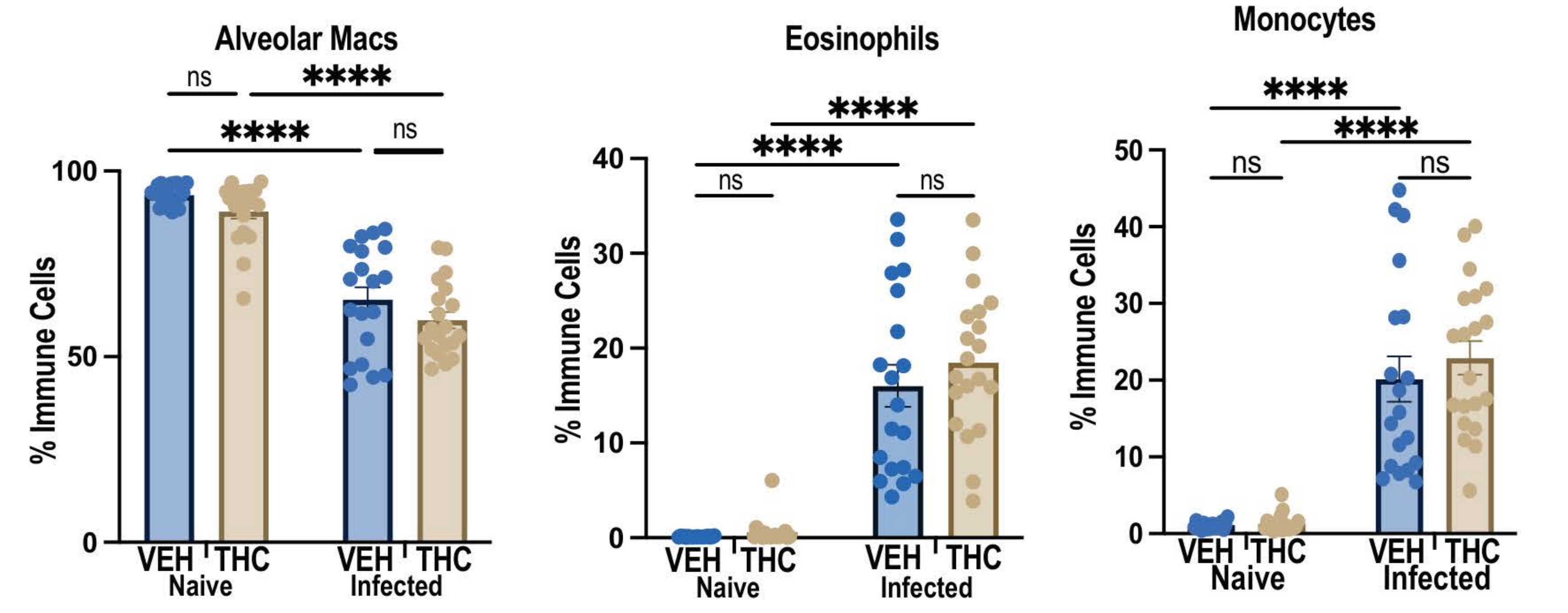

Supplemental Figure 3. Flow cytometry gating strategies for BALF immune cell analysis. (A) Gating for alveolar macrophages, eosinophils, and $CD4^+$ T cells in naïve and infected mice. (B) Frequency of alveolar macrophages, eosinophils and monocytes among $CD45^+$ cells. Data: mean ± SEM pooled from 2 experiments (n=4-7 mice/group/exp; two-way ANOVA with Tukey's test). ***p<0.001; ****p<0.0001; ns, not significant.

Sup. Fig. 4.

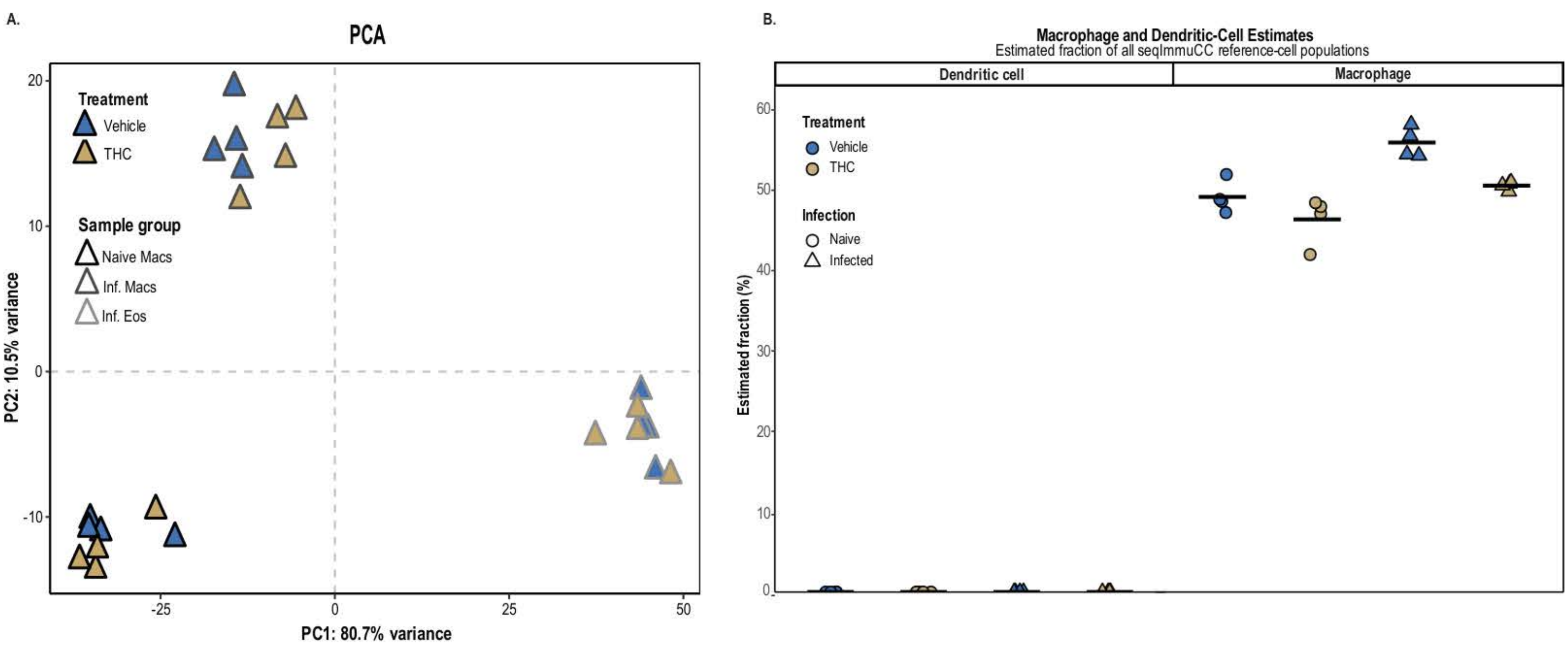

Supplemental Figure 4. PCA and deconvolution of lung RNA-seq samples. (A) PCA of variance-stabilized expression data from CD11c^{+} lung macrophage-enriched cells and CD11c^{-}Siglec-F^{+} lung eosinophils (axes show variance explained). (B) Deconvolution estimates of DC and macrophage reference populations within CD11c^{+} samples. Data: 1 experiment (n=7 mice/group pooled into 4 libraries/group).

Sup. Fig. 5.

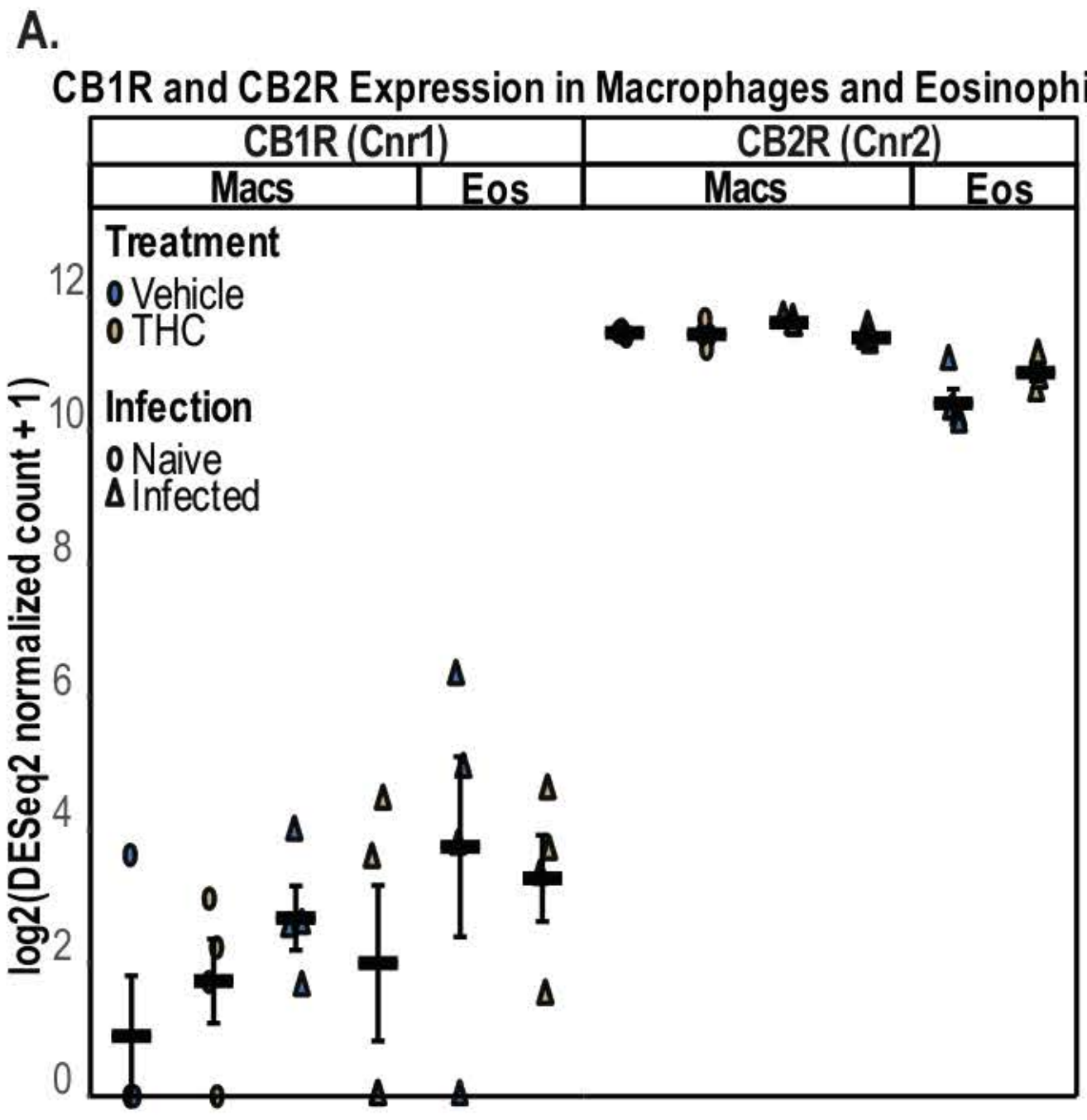

Supplemental Figure 5. Cannabinoid receptor and type 2 cytokine pathway expression in RNA-seq and ex vivo splenocyte stimulation. Targeted gene expression in $CD11c^{+}$ macrophage-enriched cells and $CD11c^{-}$Siglec-F^{+} eosinophils from vehicle- and THC-treated mice. (A) Cnr1 (CB1R) and Cnr2 (CB2R) expression. (B–G) Pathway gene expression in eosinophils and macrophage-enriched cells for eotaxin/CCR3 (Ccl11, Ccl24, Ccr3; B, C), IL-5 (Csf2rb, Il5, Il5ra; D, E), and IL-13 (Il13, Il13ra1, Il4ra, Il13ra2; F, G). (H, I) Ex vivo splenocyte stimulation concentrations of IL-4 (H) and IL-5 (I). Data: mean ± SEM. RNA-seq: 1 experiment (n=7 mice/group, pooled into 4 libraries/group). Cytokine data: pooled from 2 experiments (n=4-7 mice/group). Macrophages: two-way ANOVA with treatment comparisons within infection conditions. Eosinophils: one-way ANOVA with Benjamini–Hochberg FDR correction. Cytokines: two-way ANOVA with Tukey's test. $p<0.05$.

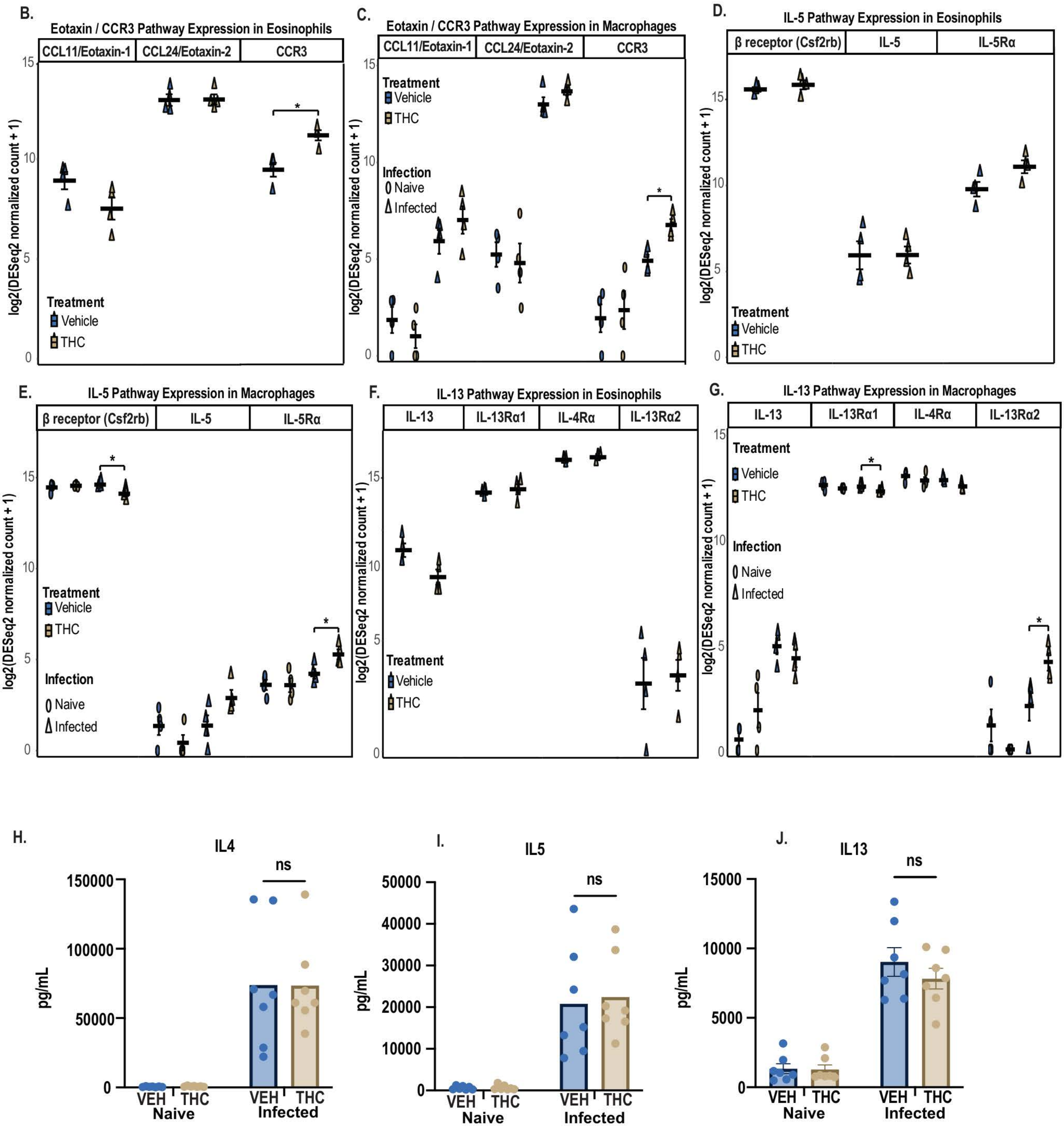

Supplement: Supplementary file 1 [file qiag131_supplementary_data.pdf]
